# Numerical Optimization of the Position in Femoral Head of Proximal Locking Screws of Proximal Femoral Nail System; Biomechanical Study

**DOI:** 10.4274/balkanmedj.2016.0732

**Published:** 2017-09-29

**Authors:** Mehmet Nuri Konya, Özgür Verim

**Affiliations:** 1 Department of Orthopeadics and Traumatology, Afyon Kocatepe University School of Medicine, Afyon, Turkey; 2 Department of Mechanics, Afyon Kocatepe University School of Engineering, Afyon, Turkey

**Keywords:** Femoral neck fractures, biomechanics, finite element analysis

## Abstract

**Background:**

Proximal femoral fracture rates are increasing due to osteoporosis and traffic accidents. Proximal femoral nails are routinely used in the treatment of these fractures in the proximal femur.

**Aims:**

To compare various combinations and to determine the ideal proximal lag screw position in pertrochanteric fractures (Arbeitsgemeinschaft für Osteosynthesefragen classification 31-A1) of the femur by using optimized finite element analysis.

**Study Design:**

Biomechanical study.

**Methods:**

Computed tomography images of patients’ right femurs were processed with Mimics. Afterwards a solid femur model was created with SolidWorks 2015 and transferred to ANSYS Workbench 16.0 for response surface optimization analysis which was carried out according to anterior-posterior (-10°<anterior-posterior<10°), inferior-superior (-6°<inferior-superior<7°) and tip-apex distance (10 mm<tip-apex distance<30 mm) proximal lag screw positions in the fracture region. The optimum position of the proximal lag screw was determined based on the von Mises stress values occurring on the fracture line. Initial analysis of the system was realized under the surgeon’s normal positioning conditions (anterior-posterior, inferior-superior=0°; tip-apex distance=12 mm).

**Results:**

The maximum and minimum (compression) von Mises stresses were found to be 438 MPa and 0.003 MPa, respectively, and risky stresses for the system occurred in the regions where the proximal lag screw passes through the proximal femoral nail hole, the small diameter portion of stem joints with a large diameter and lag screw mounts to the stem. The most suitable position of the proximal lag screw was found at the middle position of the tip-apex distance (20 mm) and femoral neck (anterior-posterior, inferior-superior=0°), according to von Mises compression stress values occurring on the fracture line.

**Conclusion:**

In our study, we couldn’t find any correlation between proximal lag screw movement and tip-apex distance on stresses of the fracture surfaces, but the proximal lag screw position in the inferior (inferior-superior<0)-superior (inferior-superior>0) and posterior-anterior directions of the femur neck significantly increased these stresses. The most suitable position of the proximal lag screw was confirmed as the middle of the femoral neck by using optimized finite element analysis.

Incidence of hip fracture is increasing continuously due to increased average age and osteoporosis (1,2,3). Biomechanical studies have enounced that intramedullary and cephalomedullary devices provide a stable structure for pertrochanteric fractures (4,5,6). Pertrochanteric fracture [Arbeitsgemeinschaft für Osteosynthesefragen (AO)-OTA 31-A1] lines (7) can start from anywhere on the greater trochanter to above or below the lesser trochanter (Figure 1). These pertrochanteric fractures may be treated with a cephalomedullary nail, sliding hip screw or plate-screw fixation (8). The ideal position of the proximal lag screw (PLS) within the femoral head to provide optimal stability to the implant-bone structure has not been studied sufficiently for cephalomedullary nails (9,10,11). Tip-apex distance (TAD) has been shown to be an important predictor for cut-out of the PLS in cephalomedullary nails (12). Although there is general consensus that the PLS in the middle of femur neck is optimal, there is continuing debate about the ideal position of the PLS (9,10,11,12,13). Our study confirms the importance of the concepts of a suitable TAD, anterior-posterior angle, and inferior-posterior angle as a clinically useful way of describing the suitable position of the PLS. In our study, we aimed to compare various combinations and to determine the ideal position of the PLS in pertrochanteric fractures of the femur by using optimized finite element analysis (FEA).

## MATERIALS AND METHODS

FEA was used to evaluate the importance of adequate positioning of the PLS within the femoral head. Initial analysis of the system was realized under the patients’ normal positioning conditions [anterior-posterior (AP), inferior-superior (IS)=0°; TAD=12 mm].

### Reconstruction of three-dimensional models from computed tomography

This study was approved by the institutional ethics committee (Afyon Kocatepe University Local ethics committee No: 2015/11/300). In our study we used femur computed tomography (CT) images of adult males with no orthopaedic disorders selected from a stored hospital imaging archive with blind selection; for this reason there was no need for patient informed consent forms. Images with 512x512 pixel resolution were obtained using a Sensation 40 CT (Siemens, Erlangen, Germany) and device adjustments were set to 120 kV and 187.5 m. CT images were processed with Mimics software (Materialise, Leuven, Belgium). A total of 665 DICOM (Digital Imaging and Communications in Medicine) images of human femurs with 1 mm slice thickness and 0.6 mm pixel size were analysed. Models were transformed into non-uniform rational basis spline surface format with SolidWorks 2015 software using point cloud methods.

Intertrochanteric femur fracture was simulated according to AO classification 31-A1 in three-dimensional (3D) modelling software (SolidWorks).

### Fea of the cephalomedullary nail

**FIG. 2.**
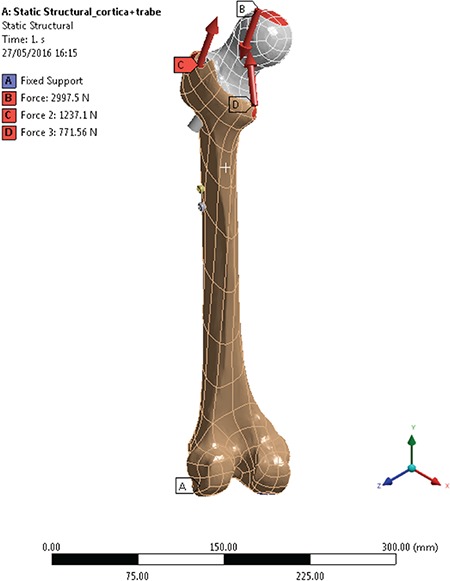
Loading condition of the femoral head.

The cephalomedullary nail on the human femur head was analysed with FEA determined under loading conditions (7,14). Material properties of all components were assigned as linear elastic isotropic. All materials of the cephalomedullary nail were made of Ti6Al4V (titanium 6, aluminium 4, vanadium alloy) with a modulus of elasticity value of 114 GPa. A value of 0.3 was assigned to all the material Poisson ratios. The modulus of elasticity of the trabecular and cortical components of the femur was taken as 0.86 GPa and 16.8 GPa, respectively (15). Our study simulated the stance phase of walking which is the most commonly used position in virtual environment studies (Figure 2). Finite element models of the proximal femoral nail (PFN) and bone system were composed of approximately 1526424 elements and 2224310 nodes. 3D 10-node tetrahedral structural solid elements were used to model the whole system. The element size was 3 mm for the cortical bone, and the contact size between the PFN and the trabecular bone was 1 mm. In this study, the main force (applied to the femoral head at 23° on the frontal and 6° on the sagittal planes) was taken to be 2460 N. Forces of the abductor and the iliopsoas muscles were taken as 1700 N (24° on the frontal and 15° on the sagittal planes) and 771 N (41° on the frontal and 26° on the sagittal planes), respectively (7,14).

### Implant design

**FIG. 3.**
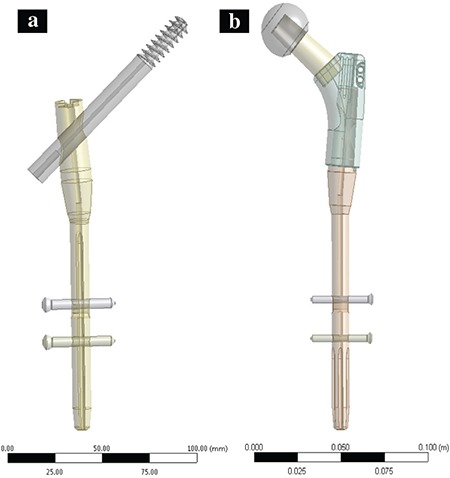
Modular nail prosthesis: (a) PFN, (b) hip prosthesis.

We used an uncemented 3D model of modular nail prosthesis combination [modular prosthesis nail combination (MNP^®^); Neologic Sağlık Hizmetleri, İzmir, Turkey] in this study. MNP is a newly designed modular hip system which combines a PFN and hip prosthesis. The femoral stem of this implant can be used as either a PFN or hip arthroplasty stem and also the can easily convert to a hip prosthesis by changing one part (Figure 3a, 3b).

### Optimization of the lag screw position

The response surface optimization method is a combination of mathematical expressions and statistical techniques used to analyse a response that is affected by several variables and to optimize the response. For many response surface methods, there is a need to estimate the mathematical forms of the functions between the response and the independent variables in the problems (16). If there is a curvature in the response surface of the system, a second order equation is needed, as follows:

A linear relationship of parameters was developed using the product moment correlation coefficient of Spearman and Pearson. A correlation-sensitivity matrix was generated to show the correlation between input and output parameters and the sensitivity of output parameters according to input parameters. The lower, initial and upper values for the input parameters used in the DOE (design of experiment) workspace are given in Table 1.

## RESULTS

In this study, the most suitable position of the lag screw and FEA of the cephalomedullary nail implanted on the human femur were examined. According to the results of the analysis of the current system, the maximum and minimum (compression) von Mises stresses were found to be 438 MPa and 0.003 MPa, respectively, and risky stresses for the system occurred in the regions where the PLS passes through the PFN hole, the small diameter portion of stem joints with a large diameter and lag screw mounts to the stem (Figure 3a, b).

**FIG. 4.**
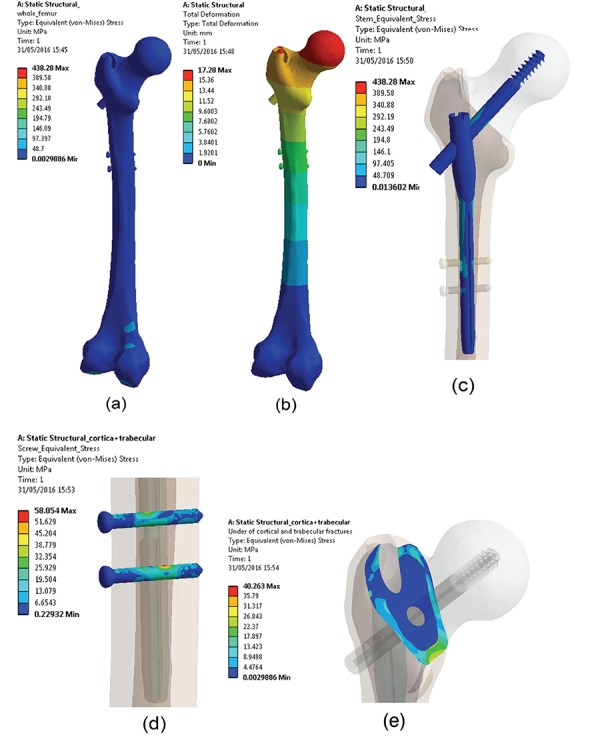
(a, b) Equivalent von Mises stress and total deformation of the cephalomedullary nail and femur, (c) stresses on the cephalomedullary nail, (d) stresses on the locking screw, (e) stresses on the fracture line of the pertrochanteric fracture.

**FIG. 5.**
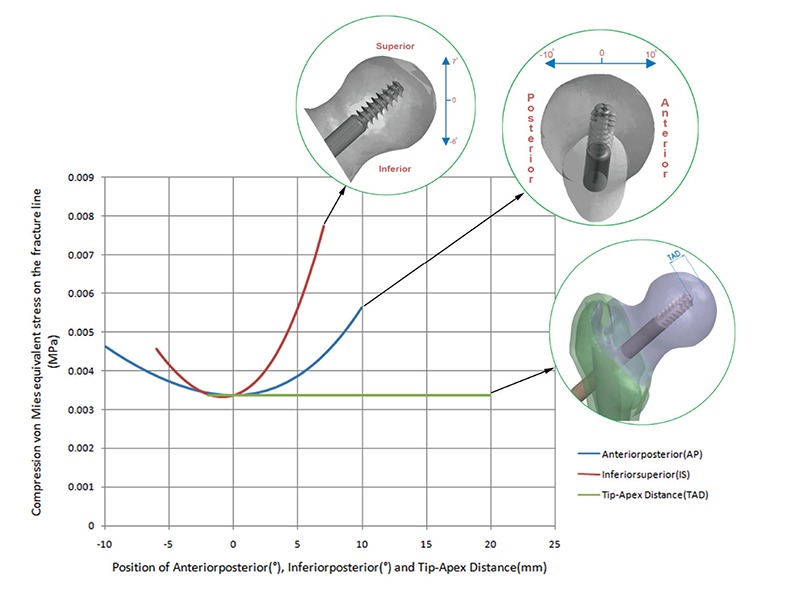
Von Mises stress distribution on the fracture line according to screw position in three planes, tip-apex distance (mm), AP (°) and IS (°).

The maximum stresses on the cephalomedullary nail occurred at the start of the stem slope (Figure 4) and stress on the cephalomedullary nail was obtained as 438 MPa. The maximum stress on the PLS was 100.4 MPa in the region where the PLS passes through the PFN hole (Figure 4), but none of the stresses occurring on the components exceeded the yield strength of the materials (Figure 4c-4e). When the stresses on the fracture line were taken into consideration according to the results of the optimization, 3D diagrams of the response surface of the parameters were reported (Figure 5).

The most suitable position of the lag screw was found at the middle position of the TAD (20 mm) and femoral neck (AP, IS=0°), according to von Mises compression stress values occurring on the fracture line. In our study, we couldn’t find any correlation with PLS movement and TAD on fracture line stress distribution, but PLS positions in the inferior (IS<0)-superior (IS>0) and anterior (AP<0)-posterior (AP>0) regions of the femur neck significantly increased these stresses. The most suitable position of the PLS, confirmed by clinical experience in the literature, is in the middle of the femoral neck and TAD (9,10,11). The worst position of the PLS is in the anterior, posterior, superior region of the femoral neck. But in this context, the risk of damage of the components was not observed.

We also determined screw displacement in every direction. According to these results screw displacement in the AP direction was -15.32 mm, mediolateral direction 0.3 mm, and SI direction 0.2 mm (Figure 4b).

## DISCUSSION

Proximal femur fractures are generally treated with internal fixation or arthroplasty (3). Both treatment regimes have advantages and disadvantages. Internal fixation consists of intramedullary techniques such as PFN and extramedullary techniques such as dynamic hip screw or plate and screw fixation techniques. In either intramedullary or extramedullary techniques, the femoral head must be stabilized with screws. The proximal femoral screw position in both techniques is important for fracture healing. In our literature review for the best screw position, some previous studies were found. Some authors reported suitable screw positions correlated with clinical experience and reported the failure rate as 6-16% (17).

Screw positioning and lag screw cut-out reasons are discussed in many studies. Most of these studies are based on clinical experience and studies done retrospectively. In this study, our FEA was totally blinded and aimed to determine different location combinations such as AP, medial-lateral, and inferior-posterior positions of the lag screw.

Kane et al. (18) discussed the importance of TAD in a cadaveric study and found that the most suitable position and found no significant differences between low-centre and centre-centre screw position treatment groups but they didn’t evaluate various screw configurations in the coronal and sagittal planes and their correlation. We evaluated nearly every point of the femoral head in different directions and load bearing either of lag screw or fracture lines.

Goffin et al. (10) tried to find the most suitable PLS position in a finite element study by using nine different positions manually. Screw positions were evaluated with the lag screw and proximal bone fragment (head and neck) and the safest seemed to be inferior middle and inferior posterior positions. A navigation system improved the lag screw position according to Regling et al. (19) cadaveric study in operations carried out by less experienced and experienced surgeons and the median TAD was 12 mm and 13 mm, respectively. In another study, Hrubina et al. (9) reported 297 patients with 308 hips treated with a dynamic hip screw; the complication rate was reported as 10% and the re-operation rate as 3.9%. In our clinical experience, there are two malpositions of the PFN which can cause pull-out of the nail but no delay or non-union in the fracture line. Munemoto et al. (20) reported hip multidetector row CT in 10 elderly patients and found the highest bone microstructure quality, in the weight-bearing part, in the superior site of the femoral head. In the femoral neck the greatest bone quality was found in the inferior region. But this study was conducted on elderly patients only and didn’t evaluate healthy people, which was reported as a limitation. As reported above, bone imaging of elderly people was used in our study; we evaluated CT images from healthy persons and the position of the proximal femoral screw was determined automatically from the program. AP, IS, and TAD measurements were evaluated. In our study the safest position is at the middle position of the TAD (20 mm) and femoral neck (AP, IS=0°), according to von Mises compression stress values occurring on the fracture line.

If the lag screw is not in an adequate position, the load can cause excess stress and this can lead to the failure of components. But, according to results of our study, the stresses on all components did not exceed the yield stress. Our study cannot perform prospectively any exact prediction of complications. In our study, according to optimized FEA, the most suitable position was found to be the middle position of the TAD (20 mm) and femoral neck (AP, IS=0°), according to von Mises compression stress values occurring on the fracture line. When mechanical properties of the bone and implant of each patient are entered into the FEA, the position of the lag screw can be estimated. Future studies should consider the results of the developed FEA compared to *in vivo* and mechanical tests with different kinds of implant and, as a clinical study, the impact of the screw position on fracture healing.

There are several limitations to note in this study. This research was a biomechanical study so we couldn’t simulate the fracture healing stages. In internal fixation devices for intertrochanteric fracture, whichever is used, lag screws pull the head to the shaft of the femur for compression which can cause femoral neck shortening and lag screws slides laterally (21). Another limitation is that we couldn’t compare the results for the femurs of patients of different gender. Strengths of this study are comparing multiple screw positions in different directions in the femoral head with optimized FEA of a newly developed PFN.

In our study, we couldn’t find any correlation between PLS movement and TAD on stresses of the fracture surfaces, but the PLS position in the inferior (IS<0)-superior (IS>0) and posterior-anterior directions of the femur neck significantly increased these stresses. The most suitable position of the PLS was confirmed as the middle of the femoral neck and TAD by using optimized FEA.

## Figures and Tables

**Table 1:**
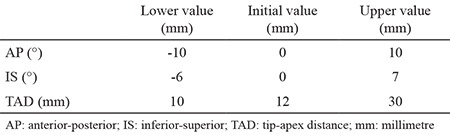
Input parameters of the proximal locking screw position

**FIG. 1.**
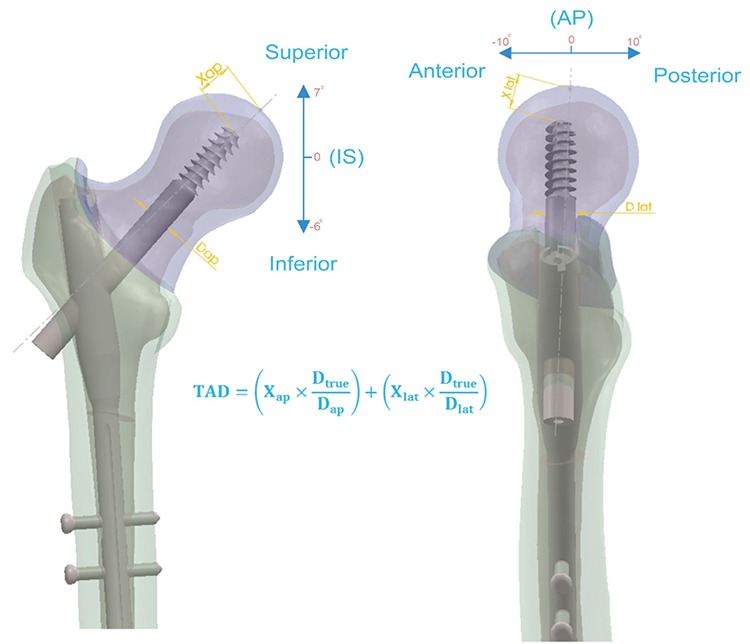
Simulation of AO 31-A1 fracture and description of tip-apex distance.
